# Skew-Laplace and Cell-Size Distribution in Microbial Axenic Cultures: Statistical Assessment and Biological Interpretation

**DOI:** 10.1155/2010/191585

**Published:** 2010-06-01

**Authors:** Olga Julià, Jaume Vidal-Mas, Nicolai S. Panikov, Josep Vives-Rego

**Affiliations:** ^1^Departament de Probabilitat, Lògica i Estadística, Facultat de Matemàtiques, Universitat de Barcelona, Gran Via, 585, 08007-Barcelona, Spain; ^2^Departament de Microbiologia, Universitat de Barcelona, Av. Diagonal, 645, 08028 Barcelona, Spain; ^3^Tres Cantos Medicines Development Campus, GlaxoSmithKline, Severo Ochoa 2, 28760 Tres Cantos, Spain; ^4^Department of Chemistry & Chemical Biology, Stevens Institute of Technology, Castle Point on Hudson, Hoboken, NJ 07030, USA

## Abstract

We report a skew-Laplace statistical analysis of both flow cytometry scatters and cell size from microbial strains primarily grown in batch cultures, others in chemostat cultures and bacterial aquatic populations. Cytometry scatters best fit the skew-Laplace distribution while cell size as assessed by an electronic particle analyzer exhibited a moderate fitting. Unlike the cultures, the aquatic bacterial communities clearly do not fit to a skew-Laplace distribution. Due to its versatile nature, the skew-Laplace distribution approach offers an easy, efficient, and powerful tool for distribution of frequency analysis in tandem with the flow cytometric cell sorting.

## 1. Introduction

Bacterial growth has been intensively studied during the last century, and the understanding of bacterial cultures has increased from decade to decade. However, the intrinsic variability and heterogeneity of bacterial axenic cultures (Vives-Rego et al. [1], Julià and Vives-Rego [2]), as well as the complexity of the different processes that take place in each phase of the growth (Prats et al. [3, 4]), still constitute a substantial difficulty when attempting to accurately study, model, and predict the bacterial growth and the resulting populations and in particular the cell size distribution. 

Flow cytometry combines direct and rapid assays to determine the number, cell-size distribution and other biochemical information regarding individual cells (Robinson [5], Shapiro [6], Vives-Rego et al. [7]). This makes it particularly attractive for studying heterogeneous bacterial populations (Davey and Kell, [8], Vives-Rego et al. [7]). Flow cytometry cell-size estimates are based on the intensity of forward light scatter (FS), which is preferred to 90° scatter or side light scatter (SS) do to its high signal intensity and its insensitivity to sub-cellular structure—conventionally described as “granulosity.” FS is generally assumed to be proportional to bacterial size (Christensen et al. [9], Julià et al. [10], Koch et al. [11], López-Amorós et al. [12]), although this relationship between particle size and FS is not monotonic as it is also affected by cell structure and chemical composition (Shapiro [6]). 

Studies on the heterogeneity of bacterial axenic cultures are scarce despite there is an obvious need to understand its morphological, biochemical, and genetic bases. The starting point in the statistical analysis of microbial heterogeneity is selecting an appropriate mathematical expression for the so-called “cumulative distribution function” for a measured parameter. More precisely, a cumulative probability distribution function is a function which gives, for each real value *x*, the probability that the measured variable takes values smaller or equal to *x*. The normal distribution (also called the Gaussian or the bell curve) remains the most commonly encountered distribution in nature and statistics due to the central limit theorem: every variable that can be modeled as a sum of many small independent variables should be normal. However, it has been clearly shown that bacterial cell size or mass distribution does not follow the Gaussian pattern (Koch [13], Vives-Rego et al. [14], Wagensberg et al. [15]). 

There is a diversity of mathematical equations used to approximate probability distributions for experimental data. They reflect to some extent the high diversity of mechanisms that exist underlying the variability observed in natural processes and biological materials. The selection of a specific mathematical model depends on the available measurement tools and the theoretical paradigm used to interpret the studied process. In any case, the type of distribution might provide important clues as to the mechanism of variability. Cell size variation is also a sensitive parameter that is influenced by the physiological and molecular-genetic state of a microbial population, for example, it can change due to the number of plasmid copies in recombinant strains (Lyncha et al. [16]) or after a temperature shock (Scherbaum [17]), osmotic stress (Elmoazzen et al. [18]) or after exposure to various pollutants (Biggs et al. [19], Ting et al. [20], Törnqvist and Claesson [21]). Therefore, the analysis of size distribution has numerous applications in biotechnological, biomedical, and environmental research.

Statistical analysis of flow cytometry data for Gram negative bacteria revealed that the skew-Laplace distribution is an effective option among other known probability functions (Julià and Vives-Rego [2]). In the present paper, we further expand this study to encompass a wider range of microorganisms including Gram-negative and Gram-positive bacteria, eukaryotic unicellular organisms (yeasts), aerobic and anaerobic species in axenic cultures or chemostat cultures as well as aquatic microbial communities containing variable mixtures of bacterial species.

We show in this paper that the skew-Laplace distribution constitutes an effective model for many axenic microbial cultures tested for scatter cytometric properties and to a lesser extend for cell size. Finally, we discuss how statistical analysis of frequency plots can be used as a tool in microbial biotechnology, cell cloning, and population dynamics studies.

## 2. Methods

### 2.1. Microbial Strains, Culture Conditions, Continuous Cultures, and Aquatic Samples

Experiments were performed with the strains listed in Table 1. Yeasts were grown in Sabouraud Maltose Broth at 26°C. Bacteria were grown in Brain and Hart Infusion medium (BHI) by incubation at 30°C. Batch cultures were inoculated with 10% of an overnight culture and shaken at 150 r.p.m. for 6, 20, or 36 hours. The chemostat cultures consisted of 250 mL flasks containing 50 mL of BHI culture permanently mixed by magnetic stirring and incubated at 30°C. The growth was started with a 2% inoculum of an overnight batch culture. The fresh medium input fluxes as well as the overflow output were controlled by a peristaltic pump (Minipuls S3, Gilson). Growth was monitored by recording the optical density at 600 nm. Once the steady-state was reached, samples were taken for the flow cytometric and electric sizing assessment. The Laplace fitting was also applied to the microscopic bacterioplankton size data from Lake Tanganyika (Africa) kindly provided by S. Pirlot and P. Servais (Pirlot et al. [22]) as well as from the coastal Cantabric Sea (Spain) (Latatu [23]).

### 2.2. Flow Cytometric Analysis

Flow cytometric experiments were carried out using an Epics XL flow cytometer (Coulter Corporation, Miami, Florida). Excitation of the sample was done using a standard 488 nm air-cooled argon-ion laser at 15 mW power. The instrument was set up with the standard configuration. The forward scatter (FS) sensor is a photodiode that collects the forward scatter, which is the laser light scattered at narrow angles (typically 2°–11°) to the axis of the laser beam. When light reaches it, the FS sensor generates voltage pulse signals proportional to the amount of light the sensor receives. Sensitivity of the flow cytometer is sufficient to detect 0.5 *μ*m particles. The side scatter (SS) is a photo diode sensor that collects the amount of laser light (488 nm) scattered at an approximate 90° angle to the axis of the laser beam. The amount of SS is proportional to the granularity of the cell that scattered the laser light. In our experiments, optical alignment was based on an optimized signal from 10 nm fluorescent beads (Flowcheck, Coulter Corporation, Miami, Florida, USA). Forward light scatter signal intensity is strongly affected by the wavelength of light used and by the precise range of angles over which light is collected, the latter being determined by focal lengths and the numerical apertures of the collecting lenses, including the size, shape, and position of irises, slits, and obscuration bars in the optical system. Since no two manufacturers of flow cytometers use the same optical design for forward scatter measurements, it is unlikely that exactly the same results will be obtained from measuring the same cells with different instruments. Theory predicts, and experiments confirm (Julià et al. [10]), that, even for uniform particles, forward scatter amplitude will not be a monotonic function of particle size. Data were analysed with WinMDI version 2.5 Software (Windows Multiple Document Interface, a flow cytometry application. Build # 05 03-09-1999, copyright 1993–98 Joseph Trotter, The Scripps Research Institute).

### 2.3. Cell Size Determination

Cell sizes were determined with a Multisizer II electronic particle analyser (Coulter Corporation), with an aperture tube of 30 *μ*m in diameter and a capacity to process 100 *μ*L of the cell suspension in 0.9% NaCl previously filtered through 0.2 *μ*m. Three types of size measurement were obtained after the transformation of the electric pulses generated by the counter: diameter, volume, and revolution surface. Data were analyzed with by AccuComp software version 1.15 (Coulter Corporation). Files were analysed with WinMDI version 2.5 Software (Windows Multiple Document Interface, a flow cytometry application. Build # 05 03-09-1999, copyright 1993–98 Joseph Trotter, The Scripps Research Institute).

### 2.4. Statistical Theory and Models

The probability model for the biomass distribution of a bacterial culture in exponential phase proposed by Wagensberg et al. [15] was a discrete model with length intervals Δ*m*. The probability *p*(*m*_*i*_) that a bacteria in the culture belongs to a class of biomass *m*_*i*_ which can be written as *m*_*i*_ = *m*_0_ + *i*Δ*m*, can be expressed as

(1) $$p\left(m_{i}\right)=\frac{1}{Z}{\left(m_{i}-m_{0}\right)}^{\gamma }\exp\;\left(-\beta m_{i}\right),$$

when *m*_0_ represents the smallest possible size required for any bacteria to exist, *γ* ≥ 0 and *β* ≥ 0 are parameters, and *Z* is the appropriate constant to make the sum of all probabilities equal to one.

We note that this model is just a discretisation of a gamma distribution scaled in *m*_0_, that has density

(2) $$f\left(m\right)=\frac{1}{U}{\left(m-m_{0}\right)}^{\gamma }\exp\;\left(-\beta m\right)\;\forall m\geq m_{0},$$

when *U* (*U* = exp (−*β* *m*_0_)Γ(*γ* + 1)*β* ^−*γ*−1^) is the necessary constant to make the integral one. 

The gamma model does not seem appropriate for our data because this distribution has always a positive skewness (2/(*γ* + 1)), whereas in bacteria context some negative skewness have been reported (see below).

Wagensberg et al. [15] deduced the mathematical expression (1) for bacteria biomass distribution using the Maximum Entropy principle. The values of constants *γ* and *β* in (1) are determined according to two constraints, the mean value and the maximization of the new born system's entropy.

One reason to use the skew-Laplace model for flow cytometer and Multisizer data is its maximum entropy property. The entropy of a probability distribution with density *f* is defined as

(3) $$\int_{-\infty }^{+\infty }\ln\;\;\left[f\left(x\right)\right]f\left(x\right)dx.$$

It is shown in (Kotz et al. [24]), that among all continuous distributions on (−*∞*, *∞*) with a given mean and first absolute moment (centered both at *μ*), *E*[*X* − *μ*] and *E*[|*X* − *μ*|], the skew-Laplace distribution provides the largest entropy.

We have calculated the skew-Laplace fits for cell size (obtained by Multisizer II) and scatter measurements (obtained by flow cytometry) from cultures of Gram-positive, Gram-negative, some yeasts and the two naturally occurring aquatic bacterial populations. The methods used to calculate the adequacy of the fit has been described previously (Julià and Vives-Rego [2, 25]). Previous studies have shown that the size distributions obtained by flow cytometer and other methods are not normal (Koch [13], Koch et al. [11], López-Amorós et al. [12], Vives-Rego et al. [14], Vives-Rego et al. [7], Wagensberg et al. [15]). Although other mathematical distributions have been used to assess bacterial size distributions, skew-Laplace distributions have only been tested in bacteria recently (Julià and Vives-Rego [2, 25]).

Given that our data on the tested microorganisms clearly show asymmetrical tails, it is not appropriate to fit the data to the normal distribution. 

The skew-Laplace distribution has the following density function with parameters *α* > 0, *β* > 0, and *μ*:

(4) $$f\left(x;\alpha ,\beta ,\mu \right)=\{\begin{array}{c} \frac{\alpha \beta }{\alpha +\beta }\exp\;\;\left(-\alpha \left(\mu -x\right)\right),\;\text{if}x\leq \mu ,\\ \frac{\alpha \beta }{\alpha +\beta }\exp\;\;\left(-\beta \left(x-\mu \right)\right),\;\text{if}x>\mu . \end{array}$$

The mean and variance of the skew-Laplace distribution are related to the three parameters (*μ*, *α*, and *β*) as follows:

(5) $$\text{Mean}:\mu -\frac{1}{\alpha }+\frac{1}{\beta },\;\text{Variance}:\frac{1}{\alpha^{2}}+\frac{1}{\beta^{2}}.$$

The parameter *μ* is closely related to the mean; in fact, when *α* = *β* (symmetric case), *μ* is the true mean, also known as the “location parameter” which implies that *μ* determines the location of the distribution origin. The distribution becomes more asymmetric as *α* differs more pronouncedly from *β*. The greater the *α* and *β* values, the more pointed is the distribution. Conversely, lower *α* and *β* values result in a flat distribution. If natural (Neperian) logarithms are applied, we obtain two straight lines with slopes *α* and −*β*, respectively,

(6) $$\ln\;\;f\left(x;\alpha ,\beta ,\mu \right)=\{\begin{array}{c} \ln\;\;\left(\frac{\alpha \beta }{\alpha +\beta }\right)-\alpha \mu +\alpha x,\;\text{if}x\leq \mu ,\\ \ln\;\;\left(\frac{\alpha \beta }{\alpha +\beta }\right)-\beta x+\beta \mu ,\;\text{if}x>\mu . \end{array}$$

This finding provides us with an important tool to verify whether the Laplace distribution is correct. When we plot the frequency curves using a natural logarithmic scale on the vertical axis, we obtain two straight lines if the model is correct. To estimate the parameters of the skew-Laplace distribution, we used the maximum likelihood method (Kotz et al. [24]). As proposed in our previous study (Julià and Vives-Rego [2]), we used two methods to verify the goodness of fit: graphical and numerical. Plots of data quantile versus skew-Laplace quantile (quantile-quantile plot) were obtained. Graphically, the proximity of these plots to the straight-line shows the goodness of fit. To quantify the quality of the skew-Lapace fitting, we calculated the critical size *N*_crit_, proposed by (Fieller et al. [26].) This statistic could be interpreted as the critical sample size, which would be required to detect lack of fit at the 5% level. The critical size *N*_crit_ is a statistic based on the chi-square goodness-of-fit test. The *N*_crit_ is defined as

(7) $$N_{\text{crit}}=\frac{\chi_{k-m-1;0.95}^{2}}{\sum_{i=1}^{k}{\left(r_{i}-p_{i}\left(\hat{\theta }\right)\right)}^{2}/p_{i}\left(\hat{\theta }\right)},$$

where *k* represents the number of intervals, *m* the number of estimated parameters, and *r*_*i*_ and $p_{i}\left(\hat{\theta }\right)$ are the sample proportion and the estimated skew-Laplace probability for the relevant interval, respectively. We have standardized the procedure to obtain 40 intervals for each sample, the more homogeneous the better. When the maximum likelihood estimation is used, the chi-square goodness-of-fit statistic has between *k* − 1 and *k* − *m* − 1 degrees of freedom (Chernoff and Lehmann [27]). The fact that *N*_crit_ is defined for *k* − *m* − 1 degrees of freedom, however, is irrelevant due to the presence of large numbers of intervals (40 in our case) with respect to the number of parameters (3 in our case). All computations were made using MatLab (MathWorks Inc., Natick, MA 01760-2098).

## 3. Results

### 3.1. Quality of the Fit and Visual Examination

We used three tools to evaluate the goodness of fit: the value *N*_crit_, the q-q plot and the frequency plot in logarithmic scale. The q-q plot is usual in statistics and represents the empirical quantiles versus theoretical model quantiles. When the fit is good, the q-q plot is nearly a straight line. 

 The other plot responds to the properties described in Section 2.4, where we demonstrated that if logarithms are applied to these frequencies and the model of skew-Laplace is valid, two straight lines result. 

The *N*_crit_ values calculated in this paper ranged between 0 and 4307. Such a large range implies that those criteria and circumstances used to determine whether the fit is acceptable or not must be defined. In theory *N*_crit_ values may range from 0 to infinite. Obviously large *N*_crit_ values indicate goodness of fit to the skew-Laplace distribution, while small values indicate poor goodness of fit. We propose to distribute the studied microorganisms into four classes according to the values of *N*_crit_.

1. For *N*_crit_ values higher than 900, the fit to the skew-Laplace distribution is excellent, the quantile-quantile plot showing a straight line, with two straight lines appearing when the frequencies are plotted in logarithmic scale.
2. For values of *N*_crit_ between 500 and 900, the fit is also satisfactory, with the quantile-quantile plot showing a nearly straight line, and two straight lines appearing when the frequencies are plotted in logarithmic scale.
3. For values of *N*_crit_ between 250 and 500, the fit is rather poor. However, the empirical distribution shape is similar to the skew-Laplace distribution. This can be seen when the frequency curves are plotted using a logarithmic scale on the vertical axis, we then obtain two straight lines except in the case of extreme values. In these cases the fit is deemed acceptable.
4. Finally, values of *N*_crit_ between 0 and 250 show that the skew-Laplace distribution is unacceptable.

**Figure 1:**
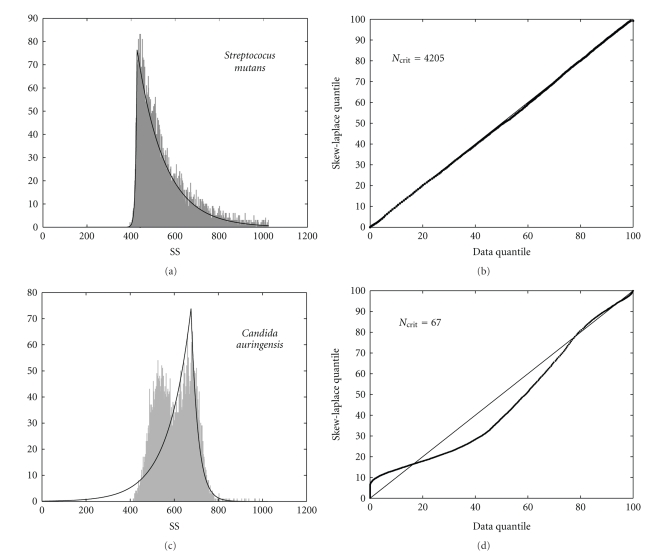
Left: skew-Laplace fit of SS from *Streptococcus mutans* and *Candida auringensis* batch culture after 24 hours of incubation. The data histogram appears in grey shadow and the continuous profile represents the estimated skew-Laplace fit. Right: Quantile plot validation (*N*_crit_ is the critical number as defined in the Section 2 and for which maxima are reported in Table 1). On the *Y*-axis are the skew-Laplace quantile and on the *X*-axis the data quantile.

**Figure 2:**
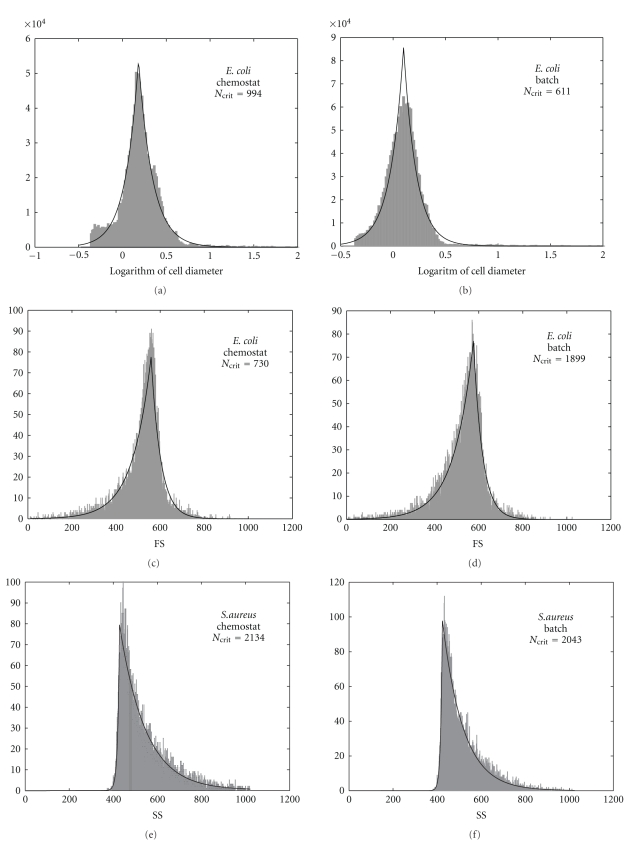
Log-skew-Laplace fit of cell size as well as skew-Laplace fit of cytometric FS and SS values. The data histogram appears in grey shadow and the continuous profile represents the estimated skew-Laplace or log-skew-Laplace fit. *E. coli* cell size (microns) in chemostat (*D* = 1.32 h^−1^) or batch culture (36 h of incubation). *E. coli* FS in chemostat (*D* = 0.96 h^−1^) or batch culture (36 h of incubation). *S. aureus* SS in chemostat (*D* = 0.96 h^−1^) or batch culture (after 20 h of incubation).

In Table 1 we have recorded the highest values that have been obtained. Based on our calculations for the strains cultured in batch or chemostat under varying conditions, we concluded not only that SS always fits the skew-Laplace distribution, but also that FS similarly fits the skew-Laplace distribution with the minor exceptions of *C. auringiensis* and *B. subtilis *(grown in chemostat). Although the cell diameter assessed by Multisizer fit the skew-Laplace distribution in many cases exceptions were observed (e.g., *S. mutans* and *S. griseus*, Table 1). Our initial conclusion is that while the FS and SS cytometric parameters follow the skew-Laplace distribution, this was not always true of the cell diameters. Figure 1 displays one of the best and one of the worst examples of fit vis-à-vis quantile validation. Figure 2 illustrates six examples of the optimal fittings for FS, SS, and cell diameter with skew-Laplace distribution shapes readily apparent, where the symmetry of the fittings is diverse. Some of the obtained fits, resulted nearly symmetrical for the logarithm of the *E. coli* cell diameter with *α* and *β* proving quite similar (*α*/*β* = 1.06 for chemostat and *α*/*β* = 0.94 for batch). We otherwise, observed a slight asymmetry for the FS of *E. coli* since the left tail is larger than the right one (negative skewness); in this case *α* is almost half of the *β* value (*α*/*β* = 0.52 for chemostat and *α*/*β* = 0.46 for batch). On the other hand, the asymmetry for the SS of *S. aureus*, was more pronounced, showing a right tail significant larger than the left one (positive skewness); in this case *α* is 12-fold greater than *β* (*α*/*β* = 12.85 for chemostat and *α*/*β* = 12.56 for batch). The absence of significant skewness differences between the studied parameters from batch and chemostat cultures are independent of the quality of their skew-Laplace distribution fit. 

### 3.2. The Skew-Laplace Fit in Cell Size and Flow Cytometric Scatters

It was found that the cell size data for microscopically measured aquatic bacteria from coastal Cantabric sea water and Tanganyika lake clearly did not fit with the skew-Laplace distribution (data not shown). This strongly suggests that the skew-Laplace fitting is a characteristic only applicable to some microbial axenic cultured populations.

Another remarkable finding is that while in our previous paper (Julià and Vives-Rego [2]), where we only analysed three Gram-negative bacteria, the skew-Laplace fitting of FS was null or poor and in our present study the fitting was acceptable. Such difference may stem from variabilities in the equipment. More specifically, the chamber of the Epics XL used in the present paper was more sensitive and generated more stable signals than the equipment used in [2] that was an Epics Elite. Another interesting aspect is that the fitting of the skew-Laplace distribution with FS is generally better than with the cell diameter, despite the assumption that flow cytometric FS values are related to bacterial size (Shapiro [6]).

The SS of the studied microbes show a strong fitting (frequently better that FS) with either the skew-Laplace or with the log-skew-Laplace distribution. Such generalized mathematical fitting suggests that an underlying biological process is at work. SS values reflect cell granulosity, which in bacteria, stems from the presence of vesicles, vacuoles, and granules of different natures (ribosome, polyphosphates, PHB, glycogen, proteins and others) (Shapiro [6]). The more ribosome and protein per bacteria, the higher is the cell's metabolic activity, cellular performance, and growth rate. In addition, the higher the growth rate, the higher is the cell concentration in proteins and ribosomes (Schaechter et al. [28]). On the other hand, granulosity caused by intracellular accumulation of reserve compounds such as lipids, starch and glycogen, is normally observed in microbial cells with retarded metabolic activity and slow growth rate (Panikov [29, 30]). Such facts strongly indicate that the metabolic activity of a culture results from different cell types exhibiting diverse metabolic activities and macromolecular content. In this case, the lower the *α* and *β* values, the higher the amplitude of the variation in metabolic activity levels.

In general at the population level, high SS values would imply a metabolically and biologically more active population than those exhibiting lower SS values. However, since we have not observed a linear relationship between increases in SS and the growth rate in chemostat experiments, such a relationship remains unclear. Since the total RNA or the ribosome cellular content is linearly related to specific growth rate, the SS values seem to be subsequently influenced by other particles inside the cell than ribosome, such as granules of lipid, starches, sulphurs, metachromatins, and so forth, (Panikov, [29, 30]). At the initial growth acceleration (e.g., growth rates between zero and 30% of maximum), the increase in ribosome content parallels a decrease in the reserve compounds. Consequently, the total granulosity and SS can remain almost constant since the ribosome increase is counterbalanced by the decrease in other granules. At higher growth rates (50%–100% of the maximum), the ribosome contribution becomes predominant, consequently, the increase in SS under accelerated growth conditions is presumably much more clearly associated with growth rate. Another possible explanation for the nonlinear correlation between the growth rate and SS is that the latter may be essentially associated with polyribosomes, since its intracellular concentration is rather low in slowly growing cells. Whether either of these two possibilities is definitively and linearly linked to SS variations, is a question that must be determined and validated by direct molecular analysis combined with flow cytometry. 

In Table 2 we present the evolution along the three incubation times of the parameters resulted from the skew-Laplace fit of FS, SS, and Multisizer values. In this table we only present three microorganisms of different morphology and we omit the log-skew-Laplace fit of FS, SS, and Multisizer values. The data presented in Table 2 come from the same replica for each of the three microorganisms on which the three FS, SS, and Multisizer values were measured at the three incubation times. When the maximum values of *N*_crit_ have been reached in another replica or for the fit of log-skew-Laplace distribution, the values reported in Table 2 do not match with those in Table 1. The presented results are only a small part of total data obtained in this study (more than 4000 values considering the replicas). Our conclusive overview of this tremendous amount of data is that there is not a clear nor repetitive trend in the skew-Laplace fit. According to our results, we cannot say that any group of microorganisms (Gram-negative, Gram-positive, or yeast) shows better fits to skew-Laplace distribution. Consequently, the observed differences seem to be the consequence of the intracellular and morphological characteristics of each strain. 

## 4. Discussion

It was not until the work of Barndorff-Nielsen and co-workers (Bagnold and Barndorff-Nielsen [31], Barndorff-Nielsen [32]) that a coherent statistical approach was formulated for the mathematical analysis of particle-size distribution. Skew-Laplace distributions were originally proposed by (Fieller et al. [26]) as a pragmatic alternative to the four-parameter hyperbolic family. Although reliable software is available for fitting hyperbolic distributions, we have ruled out this family as it is too complex and because similarity of distribution does not necessarily mean that the parameters are similar.

Since the skew-Laplace fittings in chemostat cultures are no better than in batch cultures, and since in general their *N*_crit_ values are inferior, we have to conclude that the skew-Laplace fitting cannot be linked to the biochemical or morphological heterogeneity of the cultures. This conclusion is supported by the fact that the skew-Laplace distribution clearly does not fit with the cell size of bacterial aquatic populations either from sea water or lake waters. Thus, the skew-Laplace model is an approach applicable to only axenic population studies, irrespective of their heterogeneity level. A plausible biological interpretation of the skew-Laplace fitting is that bacterial cultures share a general mathematical distribution with small, repetitive biological and nonbiological materials.

We found a satisfactory fit in many cases, most of them corresponding to the 6 h of incubation (exponential phase) and for those microorganisms exhibiting low morphological variability. In those cases were we observe a lack of the goodness of fit, the skew-Laplace model was not able to give a satisfactory quantitative measure of the differences among irregular and highly variable morphotypes.

These facts reinforce the general assumption that cells from an axenic culture are inherently heterogeneous in many aspects as recently simulated by Prats et al. [3, 4]. This includes the kinetic properties of the individual cells as shown by the ribosomal and granular density variability when indirectly assessed via SS values. Such heterogeneity is completely overlooked in most studies. 

Taking into account the high bacterial diversity observed in bacterial axenic cultures (Vives-Rego et al. [1]), t is likely that due to any environmental change, a fraction of the cell population will have a selective advantage. If so, an axenic culture exposed to various substrates will produce different final populations. Consequently, the biological properties of the resulting cultures will not be identical, despite having originated from the same axenic culture.

Simulations previously reported (Prats et al. [3, 4, 33]) showed a forward shift of the biomass distribution during the lag phase, a maintained stability during the exponential phase and a backwards shift during the transition to stationary phase. This behavior was experimentally observed and validated in an *Escherichia coli* culture using flow cytometry and particle size analysis measurements (Prats et al. [33]). These simulation results strongly indicate that bacterial cultures exhibit an intrinsic and extrinsic variability that cannot be always and perfectly adjusted to a continuous and deterministic model as the skew-Laplace distribution. Cell size distribution may change as a function of the symmetric (*E. coli* and others) or asymmetric (yeasts) type of cell, the cell division mechanism, the cell cycle phase (lag, exponential, or stationary) as well as the genetic and environmental factors.

 A genome can be accurately duplicated and clone populations can be fully homogeneous if mutations, genetic transfers, and internal genetic recombination do not drive the population towards heterogeneity. These factors are therefore, the main pillars of diversity and evolutionary potential in bacterial populations. A bacterium may express various phenotypes without a difference in genotype due to alterations in the microenvironment. 

A practical consequence of this new-found property (the skew-Laplace fit) concerning axenic cultures is that flow cytometric definitions of high granulosity subpopulations would be useful in selecting mutants (experimentally or naturally). Once the high granulosity subpopulation is cytometrically sorted, any genetic process affecting it will be more productive than in those cultures containing both high and low granulosity populations. Spontaneously and naturally induced mutations would also be more effective in exclusively high granulosity subpopulations than in conventionally mixed cultures containing both high and low granulosity populations. Any experimental or natural process within axenic cultures intended to obtain high numbers of mutations will be more successful if applied to a subpopulation with high granulosity, versus that with lower granulosity.

The main advantages of the skew-Laplace approach are: (i) it provides a simple but effective tool for analyzing distribution frequencies; (ii) the resulting graphs can be compared visually quite easily; (iii) the three numerically associated parameters of the graphs (*μ*, *α*, *β*) allow easy and rapid comparison of the quantitative differences among similar distributions; (iv) the skew-Laplace approach optimizes the potential of flow cytometric cell sorting since it provides a better mathematical delimitation of small subpopulations for subsequent cell sorting; (v) it can be easily incorporated into the standard software of automatic readers used for cells parameters in general and in flow cytometry in particular; (vi) it enlarges the palette of tools in the field of populations analyses related to microbiology, biotechnology, and eukaryotic clone studies. A final biological interpretation of the fitting is that bacterial axenic cultures share a general mathematical distribution of small, repetitive biological and nonbiological materials. This shared mathematical behavior probably also reflects a general physical law that applies to all small particles, irrespective of whether they have a biological origin.

## Figures and Tables

**Table 1:** Strains used in the present study and the highest (or maximal) *N*_crit_ values obtained.

Strain	Reference code	B/C	FS	SS	Multisizer
*Acinetobacter calcoaceticus*	CECT 441T	B	*955 (20 h)	**1260 (6 h)	*511 (20 h)
*Aeromonas hydrophyla*	CECT 5174	B	*1554 (20 h)	**1638 (6 h)	*757 (6 h)
*Alcaligenes faecalis ssp. faecalis *	CECT 145	B	*1615 (6 h)	*749 (20 h)	*513 (6 h)
*Alteromonas macleodii*	CECT 4198T	B	**459 (6 h)	*472 (36 h)	*406 (20 h)
*Arthrobacter globiformis*	CECT 388T	B	*707 (6 h)	*523 (36 h)	*197 (20 h)
*Bacillus megaterium*	CECT 44	B	**726 (20 h)	*1063 (36 h)	**112 (36 h)
*Bacillus subtilis*	CECT 35	B	**744 (36 h)	**3013 (20 h)	*809 (20 h)
*Corynebacterium variabile*	CECT 4164	B	*594 (6 h)	*432 (6 h)	**444 (20 h)
*Enterobacter aerogenes*	CECT 684T	B	*2751 (6 h)	**1186(36 h)	**247 (20 h)
*Escherichia coli*	ATCC 8731	B	*1899 (36 h)	**546 (20 h)	**611 (36 h)
*Micrococcus luteus*	CECT 243	B	**555 (36 h)	*1612 (6 h)	*734 (36 h)
*Paracoccus denitrificans*	CECT 694	B	**1485 (6 h)	*2641 (6 h)	**316 (20 h)
*Proteus mirabilis*	CECT 170	B	*936 (6 h)	**1615 (20 h)	*230 (6 h)
*Pseudomonas aeruginosa*	CECT 180	B	*4255 (6 h)	**990 (20 h)	**266 (6 h)
*Pseudomonas stutzeri*	CECT 930T	B	*467 (36 h)	*933 (6 h)	**303 (20 h)
*Salmonella choleraesuis ssp. arizonae *	CECT 4395	B	*2888 (6 h)	**582 (20 h)	**1777 (6 h)
*Staphylococcus aureus ssp. aureus*	CECT 59	B	**1770 (20 h)	*2043 (20 h)	**1346 (6 h)
*Streptococcus mutans*	CECT 479T	B	*952 (6 h)	*4205 (6 h)	0
*Streptomyces antibioticus*	CECT 3213	B	*1415 (6 h)	*4307 (6 h)	**217 (36 h)
*Streptomyces griseus sp. griseus*	CECT 3102	B	*1697 (6 h)	*642 (6 h)	0
*Vibrio fischeri*	CECT 524T	B	**533 (6 h)	*257 (6 h)	**146 (36 h)
*Xanthomonas campestris*	CECT 95	B	**795 (6 h)	*1227 (6 h)	**180 (36 h)
*Candida albicans*	CECT 1001	B	*300 (36 h)	**336 (20 h)	**301 (20 h)
*Candida auringiensis*	CECT 10611	B	**143 (20 h)	*607 (6 h)	**321 (20 h)
*Pichia guillermondii*	CECT 1019	B	**1210 (36 h)	*548 (6 h)	**187 (20 h)
*Saccharomyces cerevisiae*	CECT 1170	B	*525 (6 h)	*1290 (20 h)	**204 (6 h)
*Saccheromyces exiguus*	CECT 1206	B	**734 (20 h)	*698 (20 h)	**86 (6 h)
*Zygosaccharomyces fermentati*	CECT 10022	B	*690 (20 h)	*339 (6 h)	**1810 (36 h)
*Escherichia coli*	ATCC 8731	C	*730 (0.96 h^−1^)	**1043 (1.92 h^−1^)	*994 (1.33 h^−1^)
*Staphylococcus aureus*	CECT59	C	*2602 (0.96 h^−1^)	*2134 (0.96 h^−1^)	*285 (0.66 h^−1^)
*Bacillus subtilis*	CECT 35	C	**124 (0.006 h^−1^)	*424 (0.006 h^−1^)	**516 (0.006 h^−1^)

1 Batch cultures were obtained at 6, 20, and 36 hours of incubation while continuous cultures were obtained at dilution rates (D) ranging from 0.36 to 1.92 h^−1^. The incubation time for the bath cultures where the maximum are reached are indicated into brackets. For continuous culture, into brackets are shown the dilution rates where the maximum are obtained.

2 CECT: Colección Española de Cultivos Tipo (Spain). ATTC: American Type Culture Colection (USA).

3 The *N*_crit_ values were obtained from skew-Laplace (*) and log skew-Laplace (**) fits in the studied batch (B) and continuous (C) cultures.

**Table 2:** Evolution along the incubation time of the *μ*, *α*, *β*, and *N *_crit_ resulting from the skew-Laplace fit of FS, SS, and Multisizer values (only three microorganisms from Table 1).

FS			
	6 h	20 h	36 h
*Escherichia coli*	*μ* = 575	*μ* = 580	*μ* = 576
	*α* = 0.023	*α* = 0.025	*α* = 0.011
	*β* = −0.022	*β* = −0.028	*β* = −0.024
	*N*_crit_ = 245	*N*_crit_ = 541	*N*_crit_ = 1899
*Staphylococcus aureus *	*μ* = 565	*μ* = 575	*μ* = 521
	*α* = 0.006	*α* = 0.009	*α* = 0.010
	*β* = −0.017	*β* = −0.018	*β* = −0.013
	*N*_crit_ = 249	*N*_crit_ = 1724	*N*_crit_ = 691

*Bacillus subtilis*	*μ* = 592	*μ* = 472	*μ* = 497
	*α* = 0.008	*α* = 0.054	*α* = 0.032
	*β* = −0.023	*β* = −0.012	*β* = −0.010
	*N*_crit_ = 615	*N*_crit_ = 223	*N*_crit_ = 114

SS			
	6 h	20 h	36 h

*Escherichia coli*	*μ* = 432	*μ* = 438	*μ* = 427
	*α* = 0.071	*α* = 0.056	*α* = 0.103
	*β* = −0.024	*β* = − 0.024	*β* = −0.011
	*N*_crit_ = 201	*N*_crit_ = 242	*N*_crit_ = 278

*Staphylococcus aureus *	*μ* = 424	*μ* = 424	*μ* = 428
	*α* = 0.134	*α* = 0.133	*α* = 0.129
	*β* = −0.009	*β* = −0.011	*β* = −0.013
	*N*_crit_ = 1082	*N*_crit_ = 2043	*N*_crit_ = 1753

*Bacillus subtilis*	*μ* = 427	*μ* = 444	*μ* = 434
	*α* = 0.168	*α* = 0.119	*α* = 0.145
	*β* = −0.016	*β* = − 0.026	*β* = − 0.027
	*N*_crit_ = 246	*N*_crit_ = 280	*N*_crit_ = 2305

Multisizer			
	6 h	20 h	36 h

*Escherichia coli*	*μ* = 1.046 microns	*μ* = 1.017 microns	*μ* = 1.060 microns
	*α* = 9.537	*α* = 10.093	*α* = 8.806
	*β* = −6.461	*β* = −5.480	*β* = −5.866
	*N*_crit_ = 423	*N*_crit_ = 338	*N*_crit_ = 420

*Staphylococcus aureus *	*μ* = 0.976 microns	*μ* = 0.815 microns	*μ* = 0.750 microns
	*α* = 9.90	*α* = 18.884	*α* = 10.854
	*β* = −2.968	*β* = −2.664	*β* = −3.410
	*N*_crit_ = 361	*N*_crit_ = 163	*N*_crit_ = 135

*Bacillus subtilis*	*μ* = 0.910 microns	*μ* = 1.204 microns	*μ* = 0.898 microns
	*α* = 10.200	*α* = 11.050	*α* = 10.733
	*β* = −2.410	*β* = −2.151	*β* = −2.422
	*N*_crit_ = 93	*N*_crit_ = 146	*N*_crit_ = 348

The parameter *μ* of the skew-Laplace distribution should not be confused with the growth rate also represented by *μ*.

## References

[1] Vives-Rego J, Resina O, Comas J, Loren G, Julià O (2003). Statistical analysis and biological interpretation of the flow cytometric heterogeneity observed in bacterial axenic cultures. *Journal of Microbiological Methods*.

[2] Julià O, Vives-Rego J (2005). Skew-Laplace distribution in Gram-negative bacterial axenic cultures: new insights into intrinsic cellular heterogeneity. *Microbiology*.

[3] Prats C, López D, Giró A, Ferrer J, Valls J (2006). Individual-based modelling of bacterial cultures to study the microscopic causes of the lag phase. *Journal of Theoretical Biology*.

[4] Prats C, Giró A, Ferrer J, López D, Vives-Rego J (2008). Analysis and IbM simulation of the stages in bacterial lag phase: basis for an updated definition. *Journal of Theoretical Biology*.

[5] Robinson JP (1999). *Current Protocols in Cytometry*.

[6] Shapiro HM (2003). *Practical Flow Cytometry*.

[7] Vives-Rego J, Lebaron P, Nebe-von Caron G (2000). Current and future applications of flow cytometry in aquatic microbiology. *FEMS Microbiology Reviews*.

[8] Davey HM, Kell DB (1996). Flow cytometry and cell sorting of heterogeneous microbial populations: the importance of single-cell analyses. *Microbiological Reviews*.

[9] Christensen H, Olsen RA, Bakken LR (1995). Flow cytometric measurements of cell volumes and DNA contents during culture of indigenous soil bacteria. *Microbial Ecology*.

[10] Julià O, Comas J, Vives-Rego J (2000). Second-order functions are the simplest correlations between flow cytometric light scatter and bacterial diameter. *Journal of Microbiological Methods*.

[11] Koch AL, Robertson BR, Button DK (1996). Deduction of the cell volume and mass from forward scatter intensity of bacteria analyzed by flow cytometry. *Journal of Microbiological Methods*.

[12] López-Amorós R, Comas J, Carulla C, Vives-Rego J (1994). Variations in flow cytometric forward scatter signals and cell size in batch cultures of *Escherichia coli*. *FEMS Microbiology Letters*.

[13] Koch AL, Neidhart C (1987). The variability and individuality of the bacteria. *Escherichia coli and Salmonella typhymurium Cellular and Mollecular Biology*.

[14] Vives-Rego J, López-Amorós R, Comas J (1994). Flow cytometric narrow-angle light scatter and cell size during starvation of *Escherichia coli* in artificial sea water. *Letters in Applied Microbiology*.

[15] Wagensberg J, López D, Valls J (1988). Statistical aspects of biological organization. *Journal of Physics and Chemistry of Solids*.

[16] Lyncha HC, Argyropoulos D, Kotsarinis M, Chuen-Im S (2000). Cell size differences in plasmid-containing and plasmid-free cells of Bacillus subtilis during batch and cyclic batch culture. *Enzyme and Microbial Technology*.

[17] Scherbaum O (1956). Cell growth in normal and synchronously dividing mass cultures of *Tetrahymena pyriformis*. *Experimental Cell Research*.

[18] Elmoazzen HY, Chan CCV, Acker JP, Elliott JAW, McGann LE (2005). The effect of cell size distribution on predicted osmotic responses of cells. *Cryo-Letters*.

[19] Biggs DC, Rowland RG, O’Connors HB, Powers CD, Wurster CF (1978). A comparison of the effects of chlordane and PCB on the growth, photosynthesis, and cell size of estuarine phytoplankton. *Environmental Pollution*.

[20] Ting YP, Lawson F, Prince IG (1991). The influence of cadmium and zinc on the cell size distribution of the alga Chlorella vulgaris. *The Chemical Engineering Journal*.

[21] Törnqvist L, Claesson A (1987). The influence of aluminum on the cell-size distribution of two green algae. *Environmental and Experimental Botany*.

[22] Pirlot S, Vanderheyden J, Descy J-P, Servais P (2005). Abundance and biomass of heterotrophic microorganisms in Lake Tanganyika. *Freshwater Biology*.

[23] Latatu A (2005). *Resistencia procariótica a la depredación en sistemas acuáticos: origen e importancia*.

[24] Kotz S, Kozubowski TJ, Podgorski K (1998). *The Laplace Distribution and Generalizations*.

[25] Julià O, Vives-Rego J (2008). A microbiology application of the skew-Laplace distribution. *Statistics and Operations Research Transactions*.

[26] Fieller NRJ, Flenley EC, Olbricht W (1992). Statistics of particle size data. *Journal of the Royal Statistical Society. Series C*.

[27] Chernoff H, Lehmann EL (1954). The use of maximum likelihood estimates in *χ*
^2^ tests for goodness of fit. *The Annals of Mathematical Statistics*.

[28] Schaechter M, Ingraham JL, Neidhardt FC (2006). *Microbios*.

[29] Panikov NS (1995). *Microbial Growth Kinetics*.

[30] Panikov NS, Flickinger MC, Drew SW (1999). Kinetics, microbial growth. *Encyclopedia of Bioprocess Technology: Fermentation, Biocatalysis and Bioseparation*.

[31] Bagnold RA, Barndorff-Nielsen O (1980). The pattern of natural size distributions. *Sedimentology*.

[32] Barndorff-Nielsen O (1977). Exponentially decreasing distributions for the logarithm of particle size. *Proceedings of the Royal Society of London. Series A*.

[33] Prats C, Ferrer J, Giró A, López D, Vives-Rego J (2010). On the evolution of cell size distribution during bacterial growth cycle: Experimental observations and individual-base model simulations. *African Journal of Microbiology Research*.

